# RAB10 overexpression promotes tumor growth and indicates poor prognosis of hepatocellular carcinoma

**DOI:** 10.18632/oncotarget.15507

**Published:** 2017-02-19

**Authors:** Wei Wang, Wei-Dong Jia, Bing Hu, Yue-Yin Pan

**Affiliations:** ^1^ Department of Medical Oncology, Anhui Provincial Hospital, Anhui Medical University, Hefei 230001, PR China; ^2^ Department of Hepatic Surgery, Anhui Provincial Hospital, Anhui Medical University, Hefei 230001, PR China

**Keywords:** RAB10, hepatocellular carcinoma, oncogene, biomarker, therapeutic target

## Abstract

Hepatocellular carcinoma (HCC), one of the most common and lethal cancers worldwide, has a high recurrence rate with current treatment modalities. Identifying biomarkers for early diagnosis and discovering new sufficient molecular targets for the development of targeted therapies are urgently needed. RAB10, a member of the RAS family, has been shown to be highly expressed in HCC. However, the function of RAB10 in HCC is less studied. Here we report that RAB10 acts as an oncogene in HCC. The shRNA-mediated knockdown of RAB10 significantly reduced the proliferation of HCC cells and colony formation, induced cell cycle arrest at G0/G1 phase and increased apoptosis *in vitro*. In addition, RAB10 knockdown suppressed HCC growth in nude mice. Moreover, RAB10 silencing decreased the phosphorylation of InsR, Met/HGFR, Ron/MST1R, Ret, c-Kit/SCFR, EphA3, EphB4, Tyro3/Dtk, Axl, Tie2/TEK, VEGFR2/KDR, Akt/PKB/Rac, S6 Ribosomal Protein and c-Abl, while the phosphorylation of HSP27, p38 MAPK, Chk2 and TAK1 increased significantly. These results suggest that RAB10 regulates cell survival and proliferation through multiple oncogenic, cell stress and apoptosis pathways. More importantly, high RAB10 expression levels in HCC cells correlated with a poor prognosis in HCC patients. Therefore, our findings revealed an oncogenic role for RAB10 in the pathogenesis of HCC and that RAB10 is a potential molecular target or a biomarker for HCC.

## INTRODUCTION

Hepatocellular carcinoma (HCC), a predominant histological subtype of primary liver cancer, is the fifth deadliest and tenth most common cancer worldwide [1, 2]. Its prognosis is gloomy with a 5-year survival of 11%. Surgery (e.g. hepatectomy and liver transplantation) provides a potentially curative treatment option for HCC patients. However, the prognosis remains poor due to high potential metastasis and recurrence. Besides, the vast majority of HCC patients are diagnosed at an advanced stage at which surgical treatments are almost impossible [3, 4]. Till now, the molecular mechanisms underlying HCC pathogenesis are still poorly understood. Identification of novel prognostic biomarkers and therapeutic targets is of significance to improve the survival of HCC patients.

Targeted therapy is a therapeutic method that specifically targets certain cancer-related genes or proteins [5, 6]. Since the expression of these genes or proteins may be abnormal in specific cancers, they could be potential biomarkers for these cancers. Drugs designed against a specific target of certain cancers will affect the cancer nidus and kill cancer cells without harming neighboring non-cancerous cells [7–9]. For instance, sorafenib is a molecular-targeted agent for the treatment of HCC with Child-Pugh A liver function [10–12]. It is a multi-kinase inhibitor of tumor growth and angiogenesis, which can significantly inhibit C and B-Raf serine/ threonine kinases, vascular endothelial growth factor receptor, platelet-derived growth factor receptor, tyrosine kinases and FLT-3 (fms related tyrosine kinase 3) [12–14]. At present, very few molecular-targeted agents have been approved for the treatment of HCC. Therefore, discovering new sufficient molecular targets for the development of HCC targeted therapies are urgently needed.

RAB10 is a protein coding gene with GTP and GDP binding domains and belongs to the RAS superfamily of small GTPases [15–17]. It can regulate intracellular vesicle trafficking. As a member of the RAS oncogene family, RAB10 has been shown to participate in the insulin-stimulated translocation of glucose transporter type 4 (GLUT4) in adipocytes [18], basement membrane secretion [19], and the formation and maintenance of the endoplasmic reticulum (ER) [20]. It regulates the transport of TLR4 which is important for innate immune responses [21, 22]. Besides, RAB10 is potently activated under autophagy-stimulating conditions, such as nutrient deprivation or mechanistic target of rapamycin (mTOR) inhibition [23]. Although recent study has shown that RAB10 was high expressed in some liver cancer tissue samples [24], the biological and clinical significances of RAB10 in HCC remain largely unclear.

Therefore, in the present study, we detected the expression of RAB10 in HCC cell lines and tissues. Lentivirus-mediated specific siRNA targeting of RAB10 was used to investigate the role of its silencing on the proliferation, colony formation, cell cycle progression and apoptosis of HCC cells. Intratumor delivery of RAB10 siRNA on xenograft tumorigenesis was also examined. Global gene profiling revealed that RAB10 regulated cell survival and proliferation through multiple oncogenic, cell stress and apoptosis pathways. Moreover, the prognostic significance of RAB10 expression in HCC patients was explored.

## RESULTS

### RAB10 is a putative oncogene of HCC

**Figure 1:**
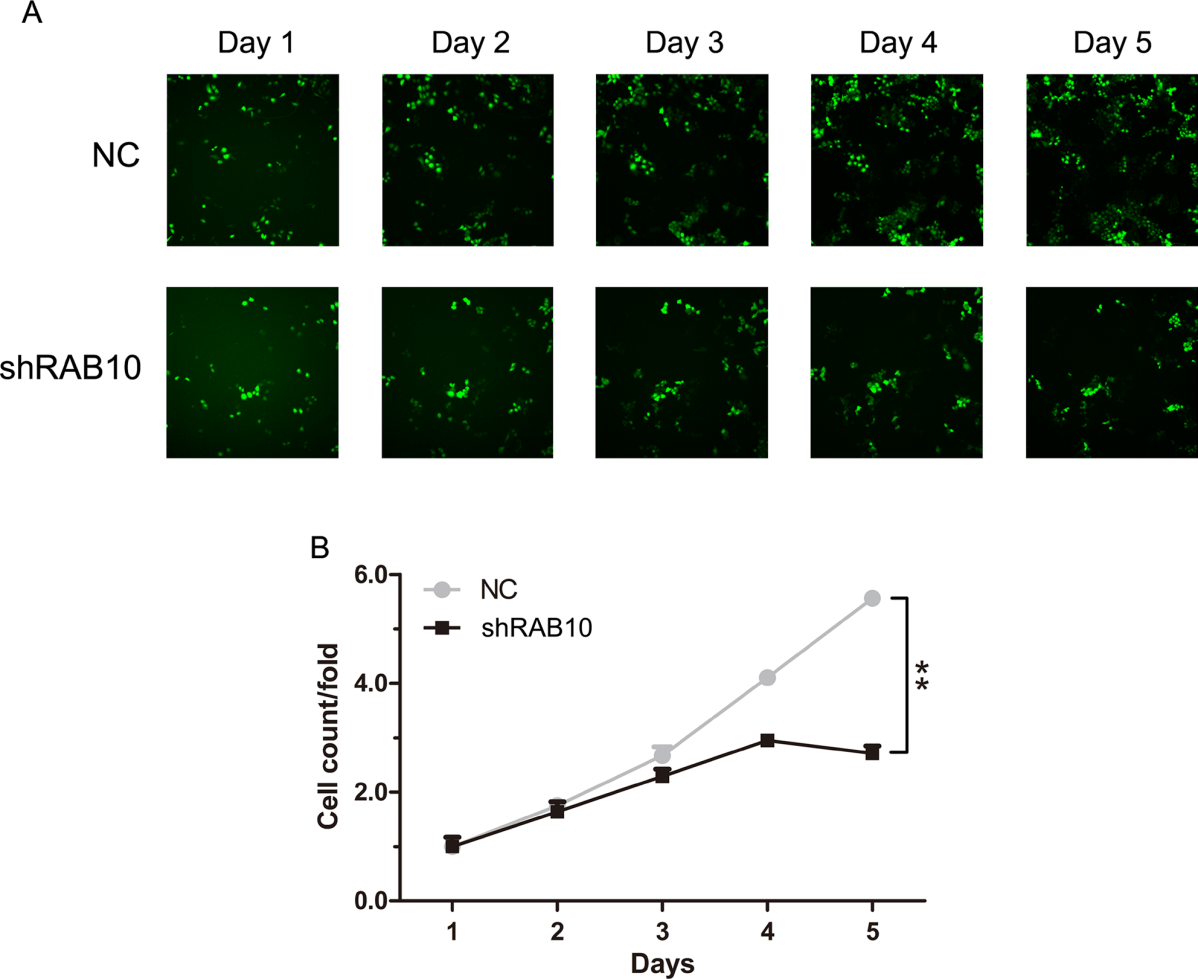
RAB10 knockdown suppressed cell proliferation in SMMC-7721 cells NC, cells infected with non-targeting shRNA lentivirus; RAB10, cells infected with RAB10-targeting shRNA lentivirus. (**A**) Cell growth was measured via multiparametric high-content screening (HCS) every day for five days after lentivirus infection. (**B**) SMMC-7721 cell numbers were quantified by Cellomics ArrayScan VTI every day and the cell proliferation rate was analyzed. Data were shown as means ± SD (*n* = 3), **P < 0.01.

A lentiviral approach mediated knockdown strategy following HCS assay (high-content screening) was performed to identify potential oncogenes in HCC. Twenty candidate genes (including GMFB, GTPBP4, RNASEH2A, UBE2S, EDIL3, SPAG5, CPSF6, RAB10, TROAP, CACYBP, FAM71D, SAE1, MLLT11, CHAF1A, POLR3C, BTG3, CEP68, MTHFD2, CHN1 and MRAP2) were chosen for the HCS assay which were highly expressed in SMMC-7721 cells. The cells maintained in good condition after the knockdown of genes. The infection efficiency was above 85% for both RAB10-siRNA lentivirus and Negative Control lentivirus (Figure 1A). Cell growth rates were defined as: cell count of the Nth day/ cell count of the 1st day, where *n* = 2, 3, 4, 5. After 5 days of culture, the knockdown of RAB10 resulted in significant decrease in both the total number of cells and the cell growth rate (Figure 1B). Compared to those infected with NC lentivirus, the cell numbers infected with shRAB10 lentivirus were 2.05-fold reduced. Thus, RAB10, an important member of the RAS family, was chosen for further study in HCC.

### Knockdown of RAB10 induced HCC cell cycle arrest and apoptosis and inhibited colony formation

**Figure 2:**
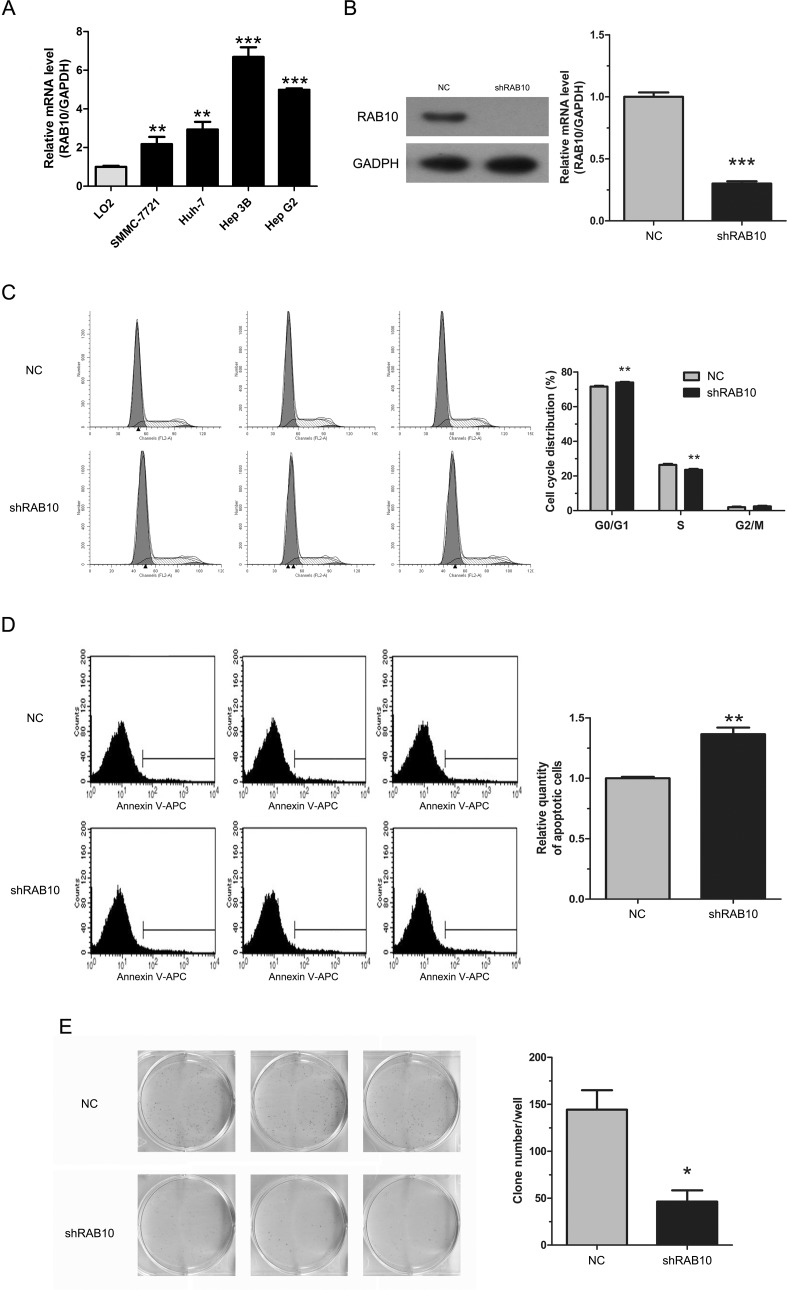
RAB10 knockdown induced cell cycle arrest and apoptosis and reduced colony formation in SMMC-7721 cells Control-shRNA, cells infected with negative control shRNA lentivirus; shRAB10, cells infected with RAB10 shRNA lentivirus. (**A**) RAB10 mRNA expression levels were detected by qRT-PCR in human normal hepatic cell line (LO2) and four HCC cell lines (SMMC-7721, Huh-7, Hep 3B and Hep G2). (**B**) RAB10 protein levels in SMMC-7721 cells and RAB10 mRNA levels in SMMC-7721 cells were knocked down efficiently by shRNA. (**C**) Knockdown of RAB10 expression induced G0/G1 phase arrest. (**D**) Knockdown of RAB10 increased apoptosis. (**E**) Knockdown of RAB10 reduced colony formation. Data were shown as means ± SD (*n* = 3), *P < 0. 05, **P < 0.01, ***P < 0.001.

We examined RAB10 expression in SMMC-7721, Huh-7, Hep-3B and HepG2 cell lines by qRT-PCR. All four HCC cell lines showed consistently high levels of expression (Figure 2A). To confirm the shRNA efficacy, protein levels were assessed by Western blot in SMMC-7721 cells. The result showed that RAB10 protein level was sharply knocked down by shRNA compared with the control (Figure 2B). Similarly, qRT-PCR revealed that the mRNA levels of RAB10 were 60% and 99% less in shRAB10-infected SMMC-7721 and HepG2 cells, respectively, than those in cells infected with NC lentivirus (Figure 2B and Supplementary Figure 1A).

Cell proliferation alteration is usually caused by changing cell cycle or apoptosis. To further explore these mechanisms, we examined the cell cycle by PI/FACS and detected apoptosis by Annexin V/FACS. As shown in Figure 2C, for SMMC-7721 cells, the control group displayed the following distribution: (G0/G1 71.57%, S 26.41%, G2/M 2.01%), and the shRAB10 group: (G0/G1 72.94%, S 22.56%, G2/M 2.5%). For HepG2 cells, the control group displayed the following distribution: (G0/G1 56.34%, S 34.36%, G2/M 9.29%), and the shRAB10 group displayed the following: (G0/G1 55.5%, S 32.0%, G2/M 12.49%). Compared to the control group, shRAB10 groups displayed a significant arrest in G0/G1 phase or G2/M phase in SMMC-7721 and HepG2 cells, respectively, suggesting that cells were arrested after RAB10 gene knockdown and RAB10 was correlated strongly with cell cycle distribution (*P* < 0.05, Figure 2C and Supplementary Figure 1B).

Moreover, as shown in Figure 2D and Supplementary Figure 1C, the percentage of SMMC-7721 cells in apoptosis phase was significantly higher in the shRAB10 group compared to the control group (3.49 ± 0.14% and 2.55 ± 0.03%, respectively; *P* = 0.005). The percentage of HepG2 cells in apoptosis phase was also significantly higher in the shRAB10 group compared to the control group (16.06 ± 1.48% and 9.48 ± 0.05%, respectively; *P* = 0.006). These results suggested that RAB10 may be associated with the apoptosis of HCC cells.

We also assessed colony formation to determine whether RAB10 knockdown affects HCC cell tumorigenesis *in vitro*. Results showed that RAB10 knockdown in both SMMC-7721 and HepG2 cells caused a substantial reduction in colony formation compared to that in control cells. In SMMC-7721 cells, there were 144 ± 21 colonies in the control group compared to 46 ± 12 in the shRAB10 group and in HepG2 cells, 137 ± 9 in the control compared to 68 ± 2 in the shRAB10 group (*P* < 0.01, Figure 2E and Supplementary Figure 1D).

### Global gene expression profiling after RAB10 knockdown in HCC cells

**Figure 3:**
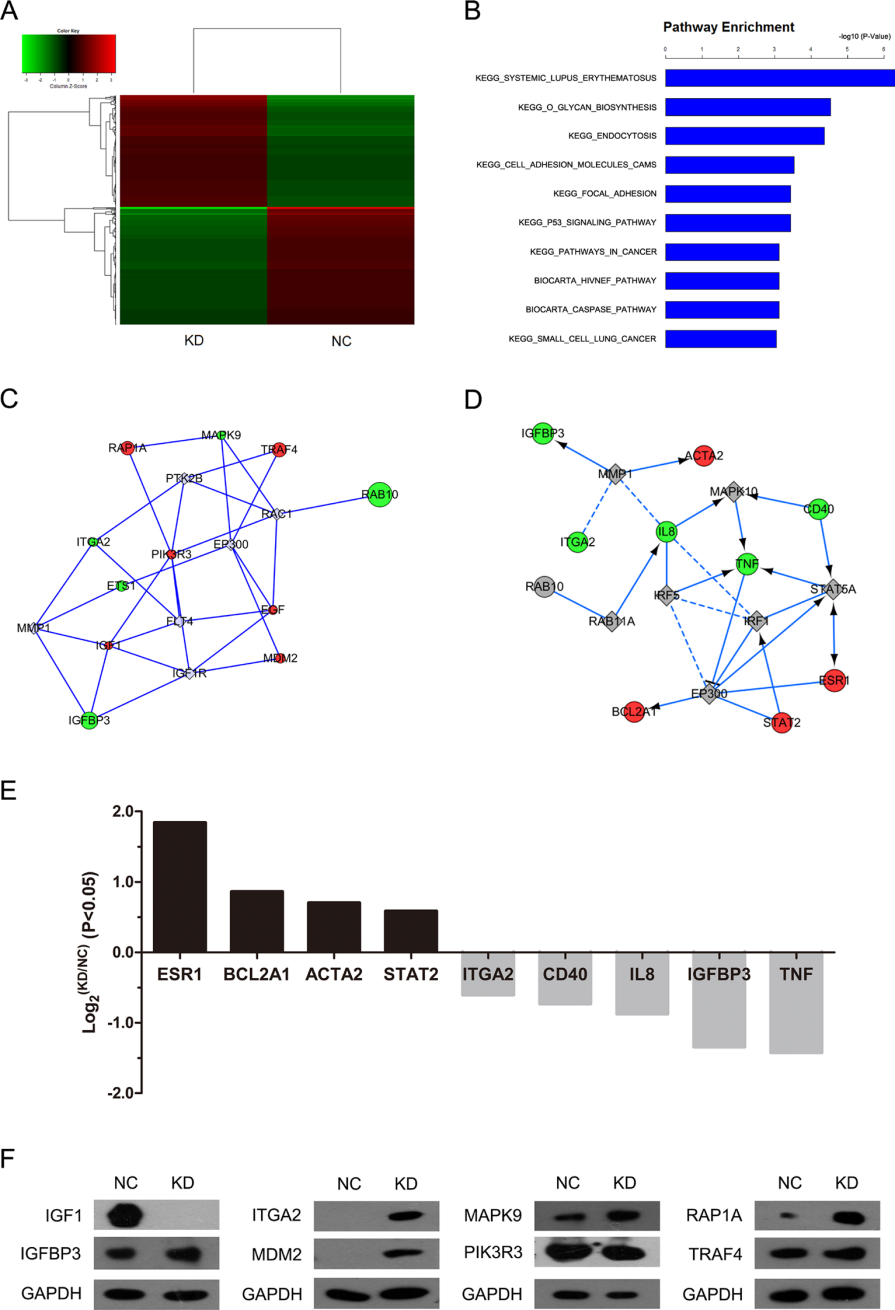
RAB10 knockdown induced global changes in SMMC-7721 gene expression NC, cells infected with negative control shRNA lentivirus; KD, cells infected with RAB10 shRNA lentivirus. (**A**) Heatmap of 695 genes showed significant (*P* < 0.05) differential expression (fold change > 1) between cells transfected with RAB10 shRNA (green) and with control shRNA (red). Rows and columns represented genes and samples, respectively. A color scale for normalized expression data was shown in the upper left corner of the heatmap (green represented down-regulated genes and red represented up-regulated genes). (**B**) Functional pathway enrichment of differentially expressed genes was analyzed based on KEGG and BIOCARTA databases. Statistically significant modulations (*P* < 0.001) of the top 10 pathways were shown. The statistical significance shown on the X axis was represented by the inverse log of the *P* value. C-D. Networks were constructed between RAB10 and select genes (**C**) and pathway-related or down-stream genes (**D**), respectively. Green circles represented down-regulated genes, red circles represented up-regulated genes, and gray rhombuses represented linker genes. Solid arrows indicated confirmed regulatory relationships and dotted lines predicted regulatory relationships. Inhibitory relationships were indicated by “T” bars. (**E**) Differentially expressed genes (*P* < 0.05 and fold change > 1) in network (D). (**F**) Protein levels of select genes in SMMC-7721 cells transfected with negative control shRNA (NC) or RAB10 shRNA (KD) measured by Western blot.

To gain insight into the mechanisms of the shRAB10-mediated tumor suppressing function, we set out to compare the transcriptome of cells infected with shRAB10 lentivirus to that of NC lentivirus-infected cells. Gene expression profiling using the Affymetrix Human GeneChip PrimeView platform identified 695 differentially expressed transcripts, based on a *P* < 0.01 threshold, in RAB10 knockdown SMMC-7721 cells compared with their control counterparts (Figure 3A). Moreover, functional analysis of the genes using GeneSpring GX software revealed that RAB10 knockdown modulated key pathways typically activated in cancer and p53 signaling (*P* < 0.001; Figure 3B, Table 1). Notably, the pathway analysis revealed that systemic lupus erythematosus signaling was the top modulated canonical pathway following RAB10 knockdown (*P* < 10^−7^; Figure 3B), and that a RAB10-mediated gene interaction network was among the most significant gene networks following topological arrangement of the differentially expressed genes by GeneSpring GX software (Figure 3C and 3D).

**Table 1:** Pathway analysis of differentially expressed genes

Gene Set Name	*P* value	Genes
KEGG_SYSTEMIC_LUPUS_ERYTHEMATOSUS	5.09E-07	15
KEGG_O_GLYCAN_BIOSYNTHESIS	2.96E-05	7
KEGG_ENDOCYTOSIS	4.29E-05	14
KEGG_CELL_ADHESION_MOLECULES_CAMS	2.96E-04	11
KEGG_P53_SIGNALING_PATHWAY	3.64E-04	8
KEGG_FOCAL_ADHESION	3.64E-04	13
BIOCARTA_CASPASE_PATHWAY	7.66E-04	5
BIOCARTA_HIVNEF_PATHWAY	7.66E-04	7
KEGG_PATHWAYS_IN_CANCER	7.66E-04	16
KEGG_SMALL_CELL_LUNG_CANCER	9.08E-04	8

In addition, we observed significant down-regulation in ITGA2, CD40, IL8, IGFBP3 and TNF, shown in the green node (Figure 3E). Meanwhile, the expression levels of ESR1, BCL2A1, ACTA2 and STAT2 were increased. The protein levels of downstream genes were detected by Western blot (Figure 3F). Our global transcriptome analysis further points to a tumor-promoting function by RAB10 in human HCC cells.

### RAB10 knockdown suppressed activation of the RTK signaling pathway and activated stress and apoptosis signaling pathways

**Figure 4:**
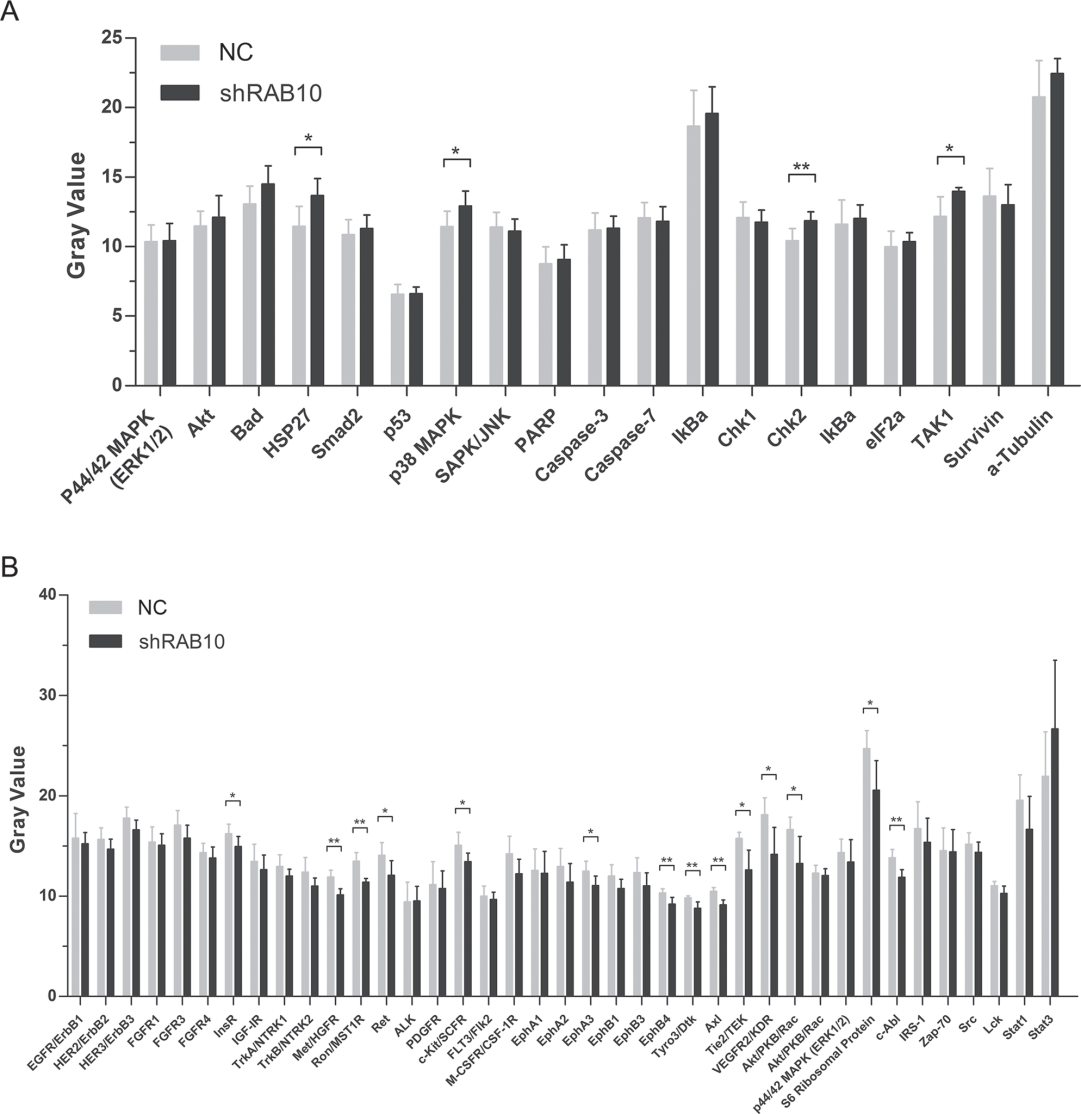
Effects of RAB10 knockdown on stress, apoptosis and RTK signaling pathways in SMMC-7721 cells shControl, cells infected with control shRNA lentivirus; shRAB10, cells infected with RAB10-targeting shRNA lentivirus. (**A**) Knockdown of RAB10 changed the expression levels of genes in stress and apoptosis signaling pathways. (**B**) Knockdown of RAB10 changed the expression levels of genes in the RTK signaling pathway. Data were shown as means ± SD (*n* = 6), *P < 0. 05, **P < 0.01, ***P < 0.001.

The abnormal expression of the RAB10-related genes was directly linked to the phenotype of tumor cell migration, invasion and metastasis. For example, RAB10 is up-regulated in HCC, and its silencing inhibited the migration and invasion of the HCC cell line AGS via the MAPK pathway. Amplified proliferation is characteristic of tumor cells, which is frequently caused by enhanced activity of intracellular signal transduction pathways. In addition, stress and apoptosis pathways are key signaling pathways involved in multiple biologic processes such as cell proliferation, differentiation, death, migration, invasion and inflammation. In order to explore the molecular mechanisms underlying RAB10-induced cell proliferation and migration, we analyzed the effect of RAB10 on oncogenic signaling pathways. PathScan results showed that the phosphorylation of InsR, Met/HGFR, Ron/MST1R, Ret, c-Kit/SCFR, EphA3, EphB4, Tyro3/Dtk, Axl, Tie2/TEK, VEGFR2/KDR, Akt/PKB/Rac, S6 Ribosomal Protein and c-Abl were reduced in SMMC-7721 cells with RAB10 knockdown. However, the elevated phosphorylation of HSP27, p38, MAPK, Chk2 and TAK1 was also observed in the cells (Figure 4A and 4B). These results suggested that activation of stress and apoptosis and inhibition of RTK signaling pathways might be responsible for RAB10 regulation of HCC cell proliferation, apoptosis and colony formation.

### RAB10 knockdown suppressed tumor growth in an SMMC-7721 xenograft mouse model

**Figure 5:**
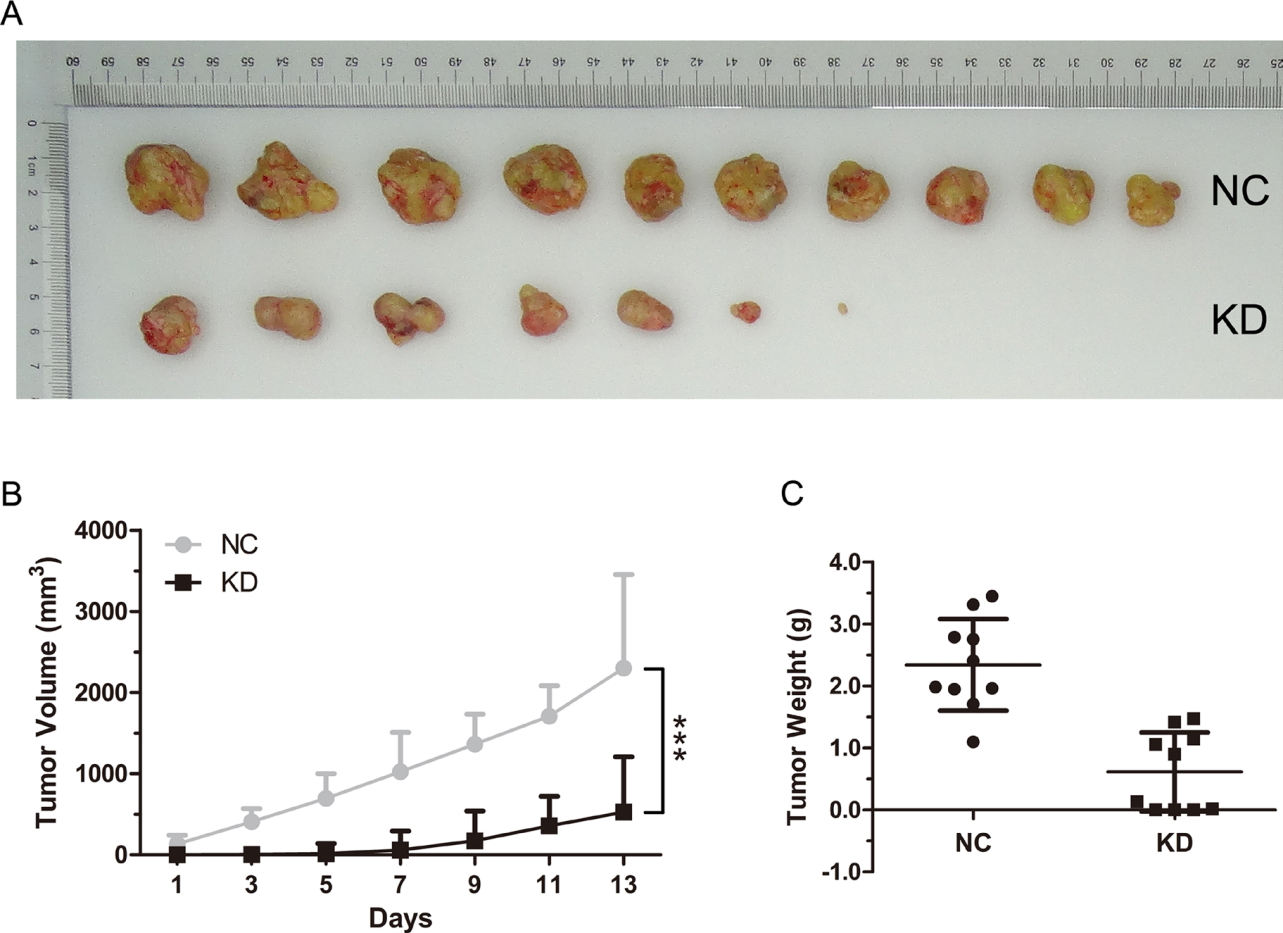
RAB10 knockdown inhibited tumorigenicity of hepatocarcinoma cells *in vivo* BALB/c nude mice were subcutaneously injected with control shRNA cells or RAB10 shRNA cells. (**A**) RAB10 knockdown significantly suppressed the formation and growth of xenografts. (**B**) Tumor volumes were measured on the indicated days. Knockdown of RAB10 decreased the volumes of xenografts significantly. (**C**) RAB10 knockdown reduced the weight of xenografts. Data were shown as means ± SD (*n* = 10), ***P < 0.001.

To examine the effects of RAB10 siRNA on tumor growth *in vivo*, SMMC-7721-RAB10 shRNA cells were transplanted into nude mice. Stably expressed shRAB10 SMMC-7721 cells with a reduction of RAB10 expression at the mRNA level (Supplementary Figure 2) or control cells (4 × 10^6^ cells per injection site) were implanted into the flanks of 6-week old female athymic mice. Control cells formed tumors in 7 days after implantation, while SMMC-7721 cells stably expressing shRAB10 failed to grow in 7 days after injection and exhibited a marked reduction in tumor size at 14 days post-implantation compared to that in control group. RAB10 shRNA treatment significantly reduced tumor size and weight compared with the control treatment (Figure 5A–5C). These data suggested that RAB10 is critical for HCC cell proliferation and tumorigenicity.

### Survival analysis and prognostic significance of RAB10 expression in HCC

**Figure 6:**
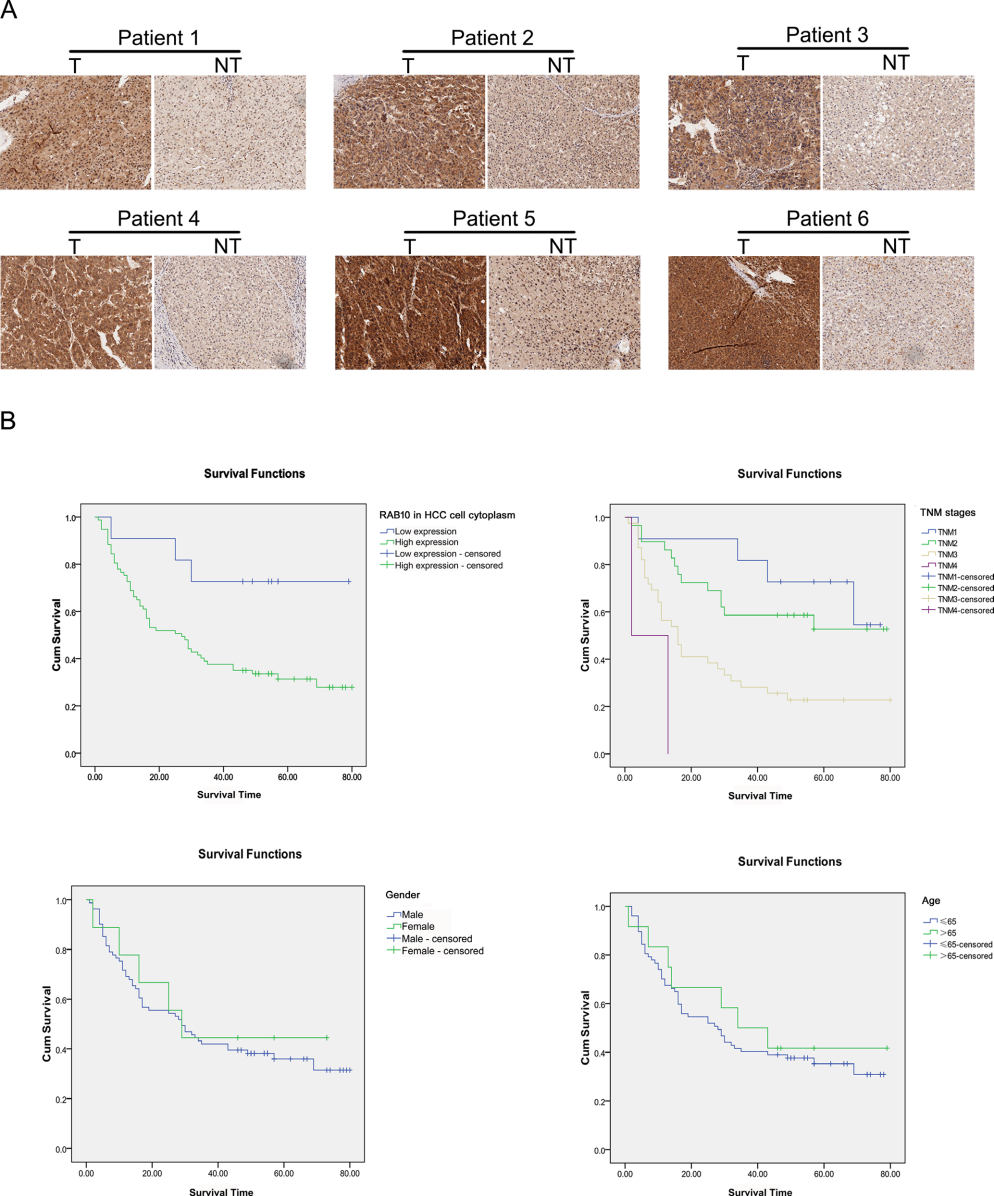
Expression of RAB10 was elevated in tissues of HCC patients (**A**) Representative pictures of immunohistochemistry analysis of RAB10 protein in each of the primary hepatocellular carcinoma tissue (T) and noncancerous tissues (NT) from the same patient. (**B**) Increased RAB10 expression is associated with shorter time to recurrence in patients with HCC. RAB10 protein expression in cytoplasm was analyzed by immunohistochemistry composed of 90 FFPE (Formalin-Fixed and Parrffin-Embedded) histologic tissue specimens. Differences in time to recurrence between patients stratified by RAB10 expression were statistically assessed by the log-rank test and Kaplan-Meier survival probability methods and depicted for TNM stage, age, gender.

Immunohistochemical analysis revealed that RAB10 staining was mainly located both in the cytoplasm and nucleus. RAB10 expression in HCC tissues was significantly higher than that in the paracarcinomatous tissues (Figure 6A, Supplementary Figure 3). We evaluated the association between RAB10 expression and clinicopathological characteristics of HCC patients, including age, gender, tumor size, pathological grading and TNM stage (Table 2). The expression level of RAB10 in HCC cell nucleus was significantly associated with the tumor size (*P* = 0.019), pathological grading (*P* = 0.003) and TNM stage (*P* = 0.027), while in paracarcinomatous cell nucleus, RAB10 was significantly associated with the pathological grading (*P* = 0.043). The expression level of RAB10 in HCC cell cytoplasm was significantly associated with pathological grading (*P* = 0.039) (Table 2). Besides, no significant correlation was found between RAB10 and other variables. Next, the Kaplan-Meier method and the log-rank test were performed to further analyze the survival rate of HCC patients with high or low RAB10 expression (Table 3). From the Kaplan-Meier survival curve, we observed that patients with high levels of RAB10 expression had significantly shorter survival time than those with low levels of RAB10 expression (*P* < 0.001) (Figure 6B). Furthermore, multivariate survival analysis conducted by Cox regression showed that high RAB10 expression in HCC cell cytoplasm was an independent prognostic factor for OS in patients with HCC after curative resection (*P* = 0.029) (Table 4). Thus, it was concluded that overexpression of RAB10 might be a novel biomarker for HCC prognosis and have important roles in HCC progression and development.

**Table 2:** Association between RAB10 expression and clinicopathological characteristics of HCC patients

	RAB10 in HCC cell cytoplasm	RAB10 in paracarcinomatous cytoplasm	RAB10 in HCC cell nucleus	RAB10 in paracarcinomatous cell nucleus
**Gender**	0.28	0.066	0.481	0.409
**Age**	0.479	0.455	0.744	0.207
**Tumor size**	0.944	0.485	0.019	0.421
**Pathological grading**	0.039	0.299	0.003	0.043
**T**	0.701	0.109	0.065	0.441
**N**	0.591	0.119	0.498	0.428
**M**	0.581	0.397	0.491	0.422
**TNM stage**	0.782	0.091	0.027	0.146

**Table 3:** Univariate survival analysis of variables associated with survival time of HCC patients

Variables	Kaplan-Meier Survival analysis Chi-Square (df)	*P*-value
**RAB10 in HCC cell cytoplasm**	5.212	0.022
**RAB10 in HCC cell nucleus**	3.203	0.073
**Gender**	0.239 (1)	0.625
**Age**	0.279 (1)	0.597
**Tumor Size (cm)**	7.778 (1)	0.005
**Pathological Grading**	1.088 (2)	0.580
**TNM stage**	20.277 (3)	0.000
**T stage**	17.876 (3)	0.000
**N stage**	1.304 (1)	0.253
**M stage**	25.675 (1)	0.000

Significant variables in the Kaplan-Meier Survival analysis were verified with log-rank statistical test.

df: different levels.

**Table 4:** Multivariate survival analysis of association between OS and clinicopathological characteristics in 90 patients with HCC

	RR	95% CI	*P* value
RAB10 in HCC cell cytoplasm	3.708	1.147–11.987	0.029
Tumor size	1.04	0.499–2.166	0.916
T	2.38	0.592–9.560	0.222
M	26.536	1.497–470.343	0.025
TNM stage	0.939	0.22–4.009	0.933

## DISCUSSION

RAB10 belongs to the RAS superfamily of small GTPases and RAB proteins, which localize to exocytic and endocytic compartments and regulate intracellular vesicle trafficking [25]. Knockdown of RAB10 decreases adhesion of cells to collagen in a HA-dependent mechanism [26] and probably also influences a number of other HA-dependent cell functions. Additionally, RAB10 may act as a multifunctional regulator of intracellular trafficking, including the secretion of proteins and cargo from the trans-Golgi network and recycling endosomes to the plasma membrane [25]. However, previous studies were focused mainly on the function of RAB10 in transport. Recent study has shown that RAB10 was high expressed in some liver cancer tissue samples [24]. But till now, the detailed information between this molecule and HCC has not been unraveled.

Therefore, in the present study, we first did a preliminarily functional characterization of RAB10 expression in HCC. Immunofluorescence analysis revealed high expression of RAB10 in HCC, and Western blot confirmed that RAB10 was expressed at higher level in human HCC tissues compared with that in the adjacent normal tissues.

Subsequently, we found that Lentivirus-mediated RAB10 knockdown markedly inhibited HCC cell proliferation *in vitro*, which was consistent with other study in osteosarcoma [27]. The total cell number and cell growth rate in shRAB10 lentivirus infected group were significantly decreased or slowed down compared with those in the NC lentivirus infected group. Colony formation assay showed RAB10 knockdown in both SMMC-7721 and HepG2 cells remarkably caused a substantial reduction in colony formation numbers. Besides, the *in vivo* tumorigenicity assay in nude mice showed that shRAB10 treatment retarded tumor growth significantly compared with that underwent the shCtrl treatment. Furthermore, RAB10 knockdown induced cell cycle arrest at G0/G1 phase or G2/M phase in SMMC-7721 and HepG2 cells, respectively and increased apoptosis. These results demonstrate that RAB10 acts as an oncogene in HCC.

Then, the phosphorylated expression levels of the RTK family members (InsR, Met/HGFR, Ron/MST1R, Ret, c-Kit/SCFR, EphA3, EphB4, Tyro3/Dtk, Axl, Tie2/TEK, VEGFR2/KDR, Akt/PKB/Rac, S6 Ribosomal Protein and c-Abl) and stress and apoptosis family members (HSP27, p38 MAPK, Chk2 and TAK1) were detected after RAB10 knockdown. And the results showed that the phosphorylation levels of RTK members were decreased and the phosphorylation levels of stress and apoptosis family members were increased. The RTK signaling pathway plays an important regulatory role in many cellular survival pathways, primarily as an inhibitor of apoptosis. Besides, it is critical for angiogenesis and tumorigenesis. Actually, combined effects of RTK and stress and apoptosis signal transduction pathways were universal in the action of antitumor agents. So our findings preliminarily suggested that RAB10 silencing may suppress HCC proliferation through inactivation of RTK signaling pathways and activation of stress and apoptosis signaling pathways. However, further studies are needed to confirm whether the activation of RTK or inactivation stress and apoptosis pathways is required for RAB10-mediated proliferation and how RAB10 regulates the phosphorylation and gene expressions.

We finally explored the prognostic significance of RAB10 in HCC. We found that RAB10 expression was significantly elevated in human HCC tissues. Moreover, the increased expression of RAB10 was positively associated with worse clinic pathologic variables, including distant metastasis and higher TNM stage, which are widely believed to be responsible for the worse prognosis noted among patients with HCC. Additionally, both univariate and multivariate survival analyses strongly demonstrated that RAB10 was a significant independent adverse prognostic factor for patients with advanced HCC, which means RAB10 could be a potential prognostic biomarker or a molecular target for HCC.

However, some limitations of this study should be noted. First, only one shRNA was used in the study and the experimental results might be interfered by the off-target effects to some extent. Second, although downstream differentially expressed genes and signaling pathways were screened out after RAB10 knockdown, the detailed regulatory mechanisms have not been elucidated fully. Finally, in clinical prognostic analysis, it was a retrospective study with relatively small sample size, which is thus associated with a potential selection bias. All these above issues will be further explored in our future studies.

In conclusion, our preliminary results suggest that RAB10 regulates cell survival and proliferation through multiple oncogenic, cell stress and apoptosis pathways. More importantly, high RAB10 expression levels in HCC cells correlated with a poor prognosis in HCC patients. Therefore, our findings revealed an oncogenic role for RAB10 in the pathogenesis of HCC and that RAB10 is a potential molecular target or a biomarker for HCC.

## MATERIALS AND METHODS

### Cell culture

Human HCC cell lines (SMMC-7721, Huh7, Hep3B and HepG2) were obtained from the Cell Bank of the China Academy of Sciences (Shanghai, China). SMMC-7721, Huh7, Hep3B and HepG2 cells were cultured in Dulbecco's modified Eagle's medium (DMEM, Gibco, Gaithersburg, USA) supplemented with 10% fetal bovine serum (FBS; HyClone Laboratories, Logan, USA), 100 U/ml penicillin and 100 μg/ml streptomycin (complete media). All cell cultures were maintained as a monolayer culture at 37°C in a humidified atmosphere containing 5% CO_2_.

### Western blot analysis

Protein samples were subjected to SDS-polyacrylamide gel electrophoresis (SDS-PAGE) and then transferred to a PVDF membrane. The membrane was blocked in Tris-buffered saline (TBS) buffer containing 0.1% Tween 20 and 5% low-fat milk for 50 min with gentle shaking. After incubation 1 hour with primary and secondary antibodies respectively, the signals were detected using the ECL chemiluminescence reagent (Millipore Corporation, Billerica, MA, USA). Protein bands were quantified by densitometric analysis using Image Lab 5.1 analysis software and levels normalized to the housekeeping protein GAPDH. The primary antibodies and secondary antibodies used were shown in Supplementary Table 1.

### Lentivirus construction and infection

To knock down RAB10 expression in cell lines, a recombinant lentiviral expression vector (pGSIL-shRAB10) containing a green fluorescent protein (GFP) tag was constructed. To generate lentiviral particles, the recombinant expression plasmid was co-transfected with a packaging plasmid system (psPAX2 and pMD2G) into SMMC-7721 cells, and viral particles were collected after 48 h. SMMC-7721 and HepG2 cells were infected with shRAB10 lentiviral vector or with a negative control (NC) vector without shRAB10 (shCon) for 24 h. The infection efficiency was preliminarily assessed in each experiment under a fluorescence microscope and then measured by sorting GFP-positive cells by flow cytometry (Beckman Coulter, USA). The stably infected cells were expanded and harvested for further experiments. The shRAB10 target sequence was as follows: GCCTTCAATACTACCTTTATT.

### Detection of intracellular signaling

To simultaneously detect a wide range of vital and well-characterized signaling molecules, cell lysates were analyzed using the PathScan^®^ Stress and Apoptosis Signaling Antibody Array Kit (Cell Signaling Technology, #7982) and the PathScan^®^ RTK Signaling Antibody Array Kit (Cell Signaling Technology, #12856) according to the manufacturer's instructions. After infection with shRAB10 or shCon for 5 days, SMMC-7721 cells were rinsed twice with ice-cold 1X phosphate-buffered saline (PBS), rapidly lysed in 1X cell lysis buffer and then incubated in Array Blocking Buffer for 20 min. An equal volume of lysate was placed in each sample and incubated for 2 h at room temperature. Before incubation with HRP-linked streptavidin, each reaction was incubated with detection antibody mixture for 1 h at room temperature. The slides were exposed to film for 25 sec after being developed with LumiGLO/Peroxide reagent (Cell Signaling Technology).

### Quantitative real-time PCR

Total RNA was extracted from SMMC-7721 or HepG2 cells using TRIzol^®^ RNA Isolation Reagent (Invitrogen, Carlsbad, CA) according to the manufacturer's instructions. Reverse transcription was performed using the PrimeScript^TM^ RT reagent kit (Takara, Dalian, China). All mRNA levels were normalized to the housekeeping gene GAPDH. The following RAB10 primers were used in this study: forward primer: 5′- TTTCACACC ATCACAACCTCC -3′, reverse primer: 5′- GGTACAAC TCTTTTGTCGTCCATA -3′. The GAPDH primers were as follows: sense primer: 5′- TGACTTCAACAGC GACACCCA -3′, antisense primer: 5′- CACCCTGTTG CTGTAGCCAAA -3′. All samples were treated under the same conditions and analyzed by qRT-PCR using SYBR Premix Ex Taq™ (Takara, Dalian, China) according to the manufacturer's protocol.

### Microarray hybridization and data analysis

The lentivirus-mediated RAB10 knockdown SMMC-7721 and control cells were collected under sterile conditions, and 1 mL TRIzol^®^ RNA Isolation Reagent (Invitrogen, Carlsbad, CA) was added to each sample. Total RNA was extracted according to the manufacturer›s recommendations. The RNA extraction and quality and integrity examinations were performed by Shanghai Genechem Co., Ltd. The quantity and quality of total RNA were assessed by NanoDrop 2000 and Agilent Bioanalyzer 2100. RNA samples were sent to examine the RNA expression profiles of the SMMC-7721 cells using the Affymetrix Gene Chip^®^ human GeneChip PrimeView Array platform (Affymetrix inc., USA). RNA samples were purified using the RNeasy kit (Qiagen, Valencia, CA) and used for the generation of biotinylated cRNA using the RNA Amplification Kit (Ambion, Austin, TX) according to the manufacturers' instructions. Individual biotinylated cRNA samples (~100 ng) were hybridized to human whole genome expression BeadChip and scanned. The quality and reproducibility of the microarray were controlled by the high levels of bead-type redundancy (up to 30 beads per probe, on average) on each array. The labeled cRNA samples were detected by hybridization to 50-mer probes on the BeadChip. After being washed and stained, the BeadChips were scanned on the GeneChip Scanner 3000.

### Colony formation assay

Cells infected with RAB10-siRNA lentivirus or NC lentivirus were seeded in 6-well plates at a density of 300–500 cells/well and further cultured in complete media for 10–15 d. After removal of the media and two rinses with PBS, the colonies were fixed with methanol for 15 min, stained with 0.1% crystal violet for 10 min and photographed using a digital camera (Leica, Germany). Experiments were repeated three times.

### Flow cytometry

SMMC-7721 and HepG2 cells infected with shRAB10 lentiviral vector or with a negative control (NC) vector without shRAB10 (shCon) were harvested at 48 hr. After double staining with FITC-Annexin V and Propidium iodide (PI) using the FITC AnnexinV Apoptosis Detection Kit (BD Biosciences) according to the manufacturer's recommendations, the cells were analyzed by flow cytometry (FACScan^®^; BD Biosciences) equipped with the CellQuest software (BD Biosciences) as previously described [28]. Cells were discriminated into viable, dead, early apoptotic or apoptotic cells, and the relative amounts of early apoptotic cells were compared to shCon. Cells for cell cycle analysis were stained with PI using the CycleTEST™ PLUS DNA Reagent Kit (BD Biosciences) following the manufacturer's protocol and analyzed by FACScan. The percentage of cells in G0/G1, S and G2/M phase were counted and compared.

### Immunohistochemistry

Clinical samples were fixed in 4% formalin overnight, dehydrated with ethanol and paraffin-embedded. Sections of 3 μm thickness were obtained using a Microm HM 355S microtome (Thermo Scientific, Ecublens, Switzerland), and tissue sections were mounted on Superfrost Plus slides (Thermo Scientific, Ecublens, Switzerland). Slides were then deparaffinized and rehydrated with xylol and alcohol. After antigen retrieval (citrate at pH 6.0 or TRIS/ EDTA at pH 9.0), sections were immunostained with anti-RAB10 primary antibody for 60 minutes and subsequently incubated with Dako EnVision HRP-conjugated secondary antibody (Catalogue No. K8000, Dako, Baar, Switzerland) for 30 minutes. In parallel, sections were counterstained with hematoxylin and eosin (H&E). Carl Zeiss Axioscope, AxioCam MRc and AxioVision 40V 4.6.3.0 software (Carl Zeiss Vision Swiss AG, Feldbach, Switzerland) were used for image acquisition and processing. Histology analysis was performed by two researchers blinded to groupings. Blood vessel count was determined in 10 representative sections of 500 × 500 μm for three different tumors of each treatment group. Percentage of tumor necrosis (lightly pink-stained surface by H&E) was quantified using the Image J (v1.46r, National Institutes of Health, USA) by analyzing 10 representative images of 3368 × 2668 μm for each condition.

### Animal experiments

Six-week-old female BALB/c nude mice were purchased from the animal center of the Cancer Institute of the Chinese Academy of Medical Science. All experimental procedures were carried out according to the National Institutes of Health guide for the care and use of Laboratory animals and complied with ARRIVE guidelines. The SMMC-7721 subcutaneous model was established as previously described [29]. The mice were randomly divided into 2 groups (10 mice per group), a RAB10 knockdown group and a mock group. The tumor size was measured with a caliper every other day, and tumor volume was calculated using the formula: volume = length × width^2^/2. At the end of a 14-day observation period, the mice were sacrificed, and tumor tissues were collected for formalin fixation and preparation of paraffin-embedded sections for immunohistochemistry.

### Human tissue specimens and tissue microarray

RAB10 protein expression was detected in HCC tissues (90 cases, collected from Anhui Provincial Hospital, Anhui Medical University) by immunohistochemical analysis. These specimens were collected and archived following protocols that were approved by the institutional review boards of the Anhui Provincial Hospital. Informed consents were obtained from all patients. 90 patients with stage I, II, III and IV HCC underwent radical resection of HCC. This group includes 81 male and 9 female with a mean age of 52.6 (range, 43–73) years. The diagnoses were conducted by at least two pathologists who were blind to the patients' information. The follow-ups were performed according to the National Comprehensive Cancer Network Practice guidelines and complied with the Declaration of Helsinki, and the end-date of the follow-ups was June 29, 2008.

### Statistical analysis

All statistical analyses were performed using SPSS 16.0 for Windows (SPSS, Inc., Chicago, IL, USA). Quantitative data were presented as mean ± standard deviation (SD). Spearman correlation coefficients (*r*) were employed to analyze the correlation between RAB10 expression and clinicopathological parameters. The Kaplan-Meier method and the log-rank test were used for survival analysis. Cox proportional hazards regression model was used for multivariate survival analysis to identify prognostic factors that were significant in the univariate analysis. A *P value* < 0.05 was considered as statistically significant.

## SUPPLEMENTARY MATERIALS TABLE AND FIGURES



## References

[1] Chen Y, Xu Y, Zhao M, Liu Y, Gong M, Xie C, Wu H, Wang Z (2016). High-throughput T cell receptor sequencing reveals distinct repertoires between tumor and adjacent non-tumor tissues in HBV-associated HCC. Oncoimmunology.

[2] Chin HG, Ponnaluri VK, Zhang G, Esteve PO, Schaus SE, Hansen U, Pradhan S (2016). Transcription factor LSF-DNMT1 complex dissociation by FQI1 leads to aberrant DNA methylation and gene expression. Oncotarget.

[3] Blechacz B, Mishra L (2013). Hepatocellular carcinoma biology. Recent Results Cancer Res.

[4] Semela D, Dufour JF (2004). Angiogenesis and hepatocellular carcinoma. J Hepatol.

[5] Daste A, Chakiba C, Domblides C, Gross-Goupil M, Quivy A, Ravaud A, Soubeyran P (2016). Targeted therapy and elderly people: A review. Eur J Cancer.

[6] Doyle JF, Forni LG (2016). Update on sepsis-associated acute kidney injury: emerging targeted therapies. Biologics.

[7] Cogdill AP, Prieto PA, Reuben A, Wargo JA (2017). Gene targeting meets cell-based therapy: raising the tail, or merely a whimper?. Clin Cancer Res.

[8] Fugle CW, Zhang Y, Hong F, Sun S, Westwater C, Rachidi S, Yu H, Garret-Mayer E, Kirkwood K, Liu B, Li Z (2016). CD24 blunts oral squamous cancer development and dampens the functional expansion of myeloid-derived suppressor cells. Oncoimmunology.

[9] Harrison JK, Van Der Wardt V, Conroy SP, Stott DJ, Dening T, Gordon AL, Logan P, Welsh TJ, Taggar J, Harwood R, Gladman JR (2016). New horizons: the management of hypertension in people with dementia. Age Ageing.

[10] Xu Z, Yang F, Wei D, Liu B, Chen C, Bao Y, Wu Z, Wu D, Tan H, Li J, Wang J, Liu J, Sun S (2016). Long noncoding RNA-SRLR elicits intrinsic sorafenib resistance via evoking IL-6/STAT3 axis in renal cell carcinoma. Oncogene.

[11] Gandhi M, Choo SP, Thng CH, Tan SB, Low AS, Cheow PC, Goh AS, Tay KH, Lo RH, Goh BK, Wong JS, Ng DC, Soo KC (2016). Single administration of Selective Internal Radiation Therapy versus continuous treatment with sorafeNIB in locally advanced hepatocellular carcinoma (SIRveNIB): study protocol for a phase iii randomized controlled trial. BMC Cancer.

[12] Parikh ND, Marshall VD, Singal AG, Nathan H, Lok AS, Balkrishnan R, Shahinian V (2017). Survival and Cost-Effectiveness of Sorafenib Therapy in Advanced Hepatocellular Carcinoma: An Analysis of the SEER-Medicare Database. Hepatology.

[13] Hsieh CH, Lin YJ, Chen WL, Huang YC, Chang CW, Cheng FC, Liu RS, Shyu WC (2017). HIF-1alpha triggers long-lasting glutamate excitotoxicity via system xc- in cerebral ischaemia-reperfusion. J Pathol.

[14] Mani SK, Zhang H, Diab A, Pascuzzi PE, Lefrancois L, Fares N, Bancel B, Merle P, Andrisani O (2016). EpCAM-regulated intramembrane proteolysis induces a cancer stem cell-like gene signature in hepatitis B virus-infected hepatocytes. J Hepatol.

[15] Vieira OV (2016). Rab3a and Rab10 are regulators of lysosome exocytosis and plasma membrane repair. Small GTPases.

[16] Isabella AJ, Horne-Badovinac S (2016). Rab10-Mediated Secretion Synergizes with Tissue Movement to Build a Polarized Basement Membrane Architecture for Organ Morphogenesis. Dev Cell.

[17] Vazirani RP, Verma A, Sadacca LA, Buckman MS, Picatoste B, Beg M, Torsitano C, Bruno JH, Patel RT, Simonyte K, Camporez JP, Moreira G, Falcone DJ (2016). Disruption of Adipose Rab10-Dependent Insulin Signaling Causes Hepatic Insulin Resistance. Diabetes.

[18] Sano H, Peck GR, Kettenbach AN, Gerber SA, Lienhard GE (2011). Insulin-stimulated GLUT4 protein translocation in adipocytes requires the Rab10 guanine nucleotide exchange factor Dennd4C. J Biol Chem.

[19] Lerner DW, McCoy D, Isabella AJ, Mahowald AP, Gerlach GF, Chaudhry TA, Horne-Badovinac S (2013). A Rab10-dependent mechanism for polarized basement membrane secretion during organ morphogenesis. Dev Cell.

[20] English AR, Voeltz GK (2013). Rab10 GTPase regulates ER dynamics and morphology. Nat Cell Biol.

[21] Barbosa MD, Johnson SA, Achey K, Gutierrez MJ, Wakeland EK, Zerial M, Kingsmore SF (1995). The Rab protein family: genetic mapping of six Rab genes in the mouse. Genomics.

[22] Chen YT, Holcomb C, Moore HP (1993). Expression and localization of two low molecular weight GTP-binding proteins, Rab8 and Rab10, by epitope tag. Proc Natl Acad Sci USA.

[23] Li Z, Schulze RJ, Weller SG, Krueger EW, Schott MB, Zhang X, Casey CA, Liu J, Stöckli J, James DE, McNiven MA (2016). A novel Rab10-EHBP1-EHD2 complex essential for the autophagic engulfment of lipid droplets. Sci Adv.

[24] He H, Dai F, Yu L, She X, Zhao Y, Jiang J, Chen X, Zhao S (2002). Identification and characterization of nine novel human small GTPases showing variable expressions in liver cancer tissues. Gene Expr.

[25] Pavlos NJ, Jahn R (2011). Distinct yet overlapping roles of Rab GTPases on synaptic vesicles. Small GTPases.

[26] Lindsay AJ, Jollivet F, Horgan CP, Khan AR, Raposo G, McCaffrey MW, Goud B (2013). Identification and characterization of multiple novel Rab-myosin Va interactions. Mol Biol Cell.

[27] Jiang W, Liu J, Xu T, Yu X (2016). MiR-329 suppresses osteosarcoma development by downregulating Rab10. FEBS Lett.

[28] Zhang SZ (2005). Knockdown of c-Met by adenovirus-delivered small interfering RNA inhibits hepatocellular carcinoma growth *in vitro* and *in vivo*. Molecular Cancer Therapeutics.

[29] Zhang J, Lu Y, Yue X, Li H, Luo X, Wang Y, Wang K, Wan J (2013). MiR-124 Suppresses Growth of Human Colorectal Cancer by Inhibiting STAT3. PLoS One.

