# Supplementary material for: RAB10 overexpression promotes tumor growth and indicates poor prognosis of hepatocellular carcinoma
Source: Oncotarget. 2017 Feb 19;8(16):26434–47. doi: 10.18632/oncotarget.15507 (PMC5432270; doi:10.18632/oncotarget.15507)
Supplement: Supplementary file 1 [file oncotarget-08-26434-s001.pdf]

# RAB10 overexpression promotes tumor growth and indicates poor prognosis of hepatocellular carcinoma

## Supplementary Materials

**Supplementary Table 1: The information of primary and secondary antibodies used in this study**

| Name | Species | Company | Cat No. | Dilution |
|---|---|---|---|---|
| RAB10 | Mouse | Abcam | ab 104859 | 1/500 |
| RAP1A | Rabbit | Abcam | ab96223 | 1/500 |
| TRAF4 | Rabbit | Abcam | ab108991 | 1/500 |
| IGFBP3 | Goat | Abcam | ab77635 | 1/200 |
| ITGA2 | Rabbit | Abcam | ab133557 | 1/500 |
| MDM2 | Rabbit | Abcam | ab38618 | 1/200 |
| IGF1 | Rabbit | Abcam | ab131476 | 1/500 |
| MAPK9 | Rabbit | ABCAM | ab76125 | 1/200 |
| PIK3R3 | Mouse | ABCAM | ab169666 | 1/500 |
| GFP | Mouse | Santa Cruz | sc-9996 | 1/4000 |
| GAPDH | Mouse | Santa Cruz | sc-32233 | 1/5000 |
| rabbit IgG | | Santa Cruz | sc-2004 | 1:5000 |
| mouse IgG | | Santa Cruz | sc-2005 | 1:5000 |
| Goat IgG | | Santa Cruz | sc-2005 | 1:2000 |

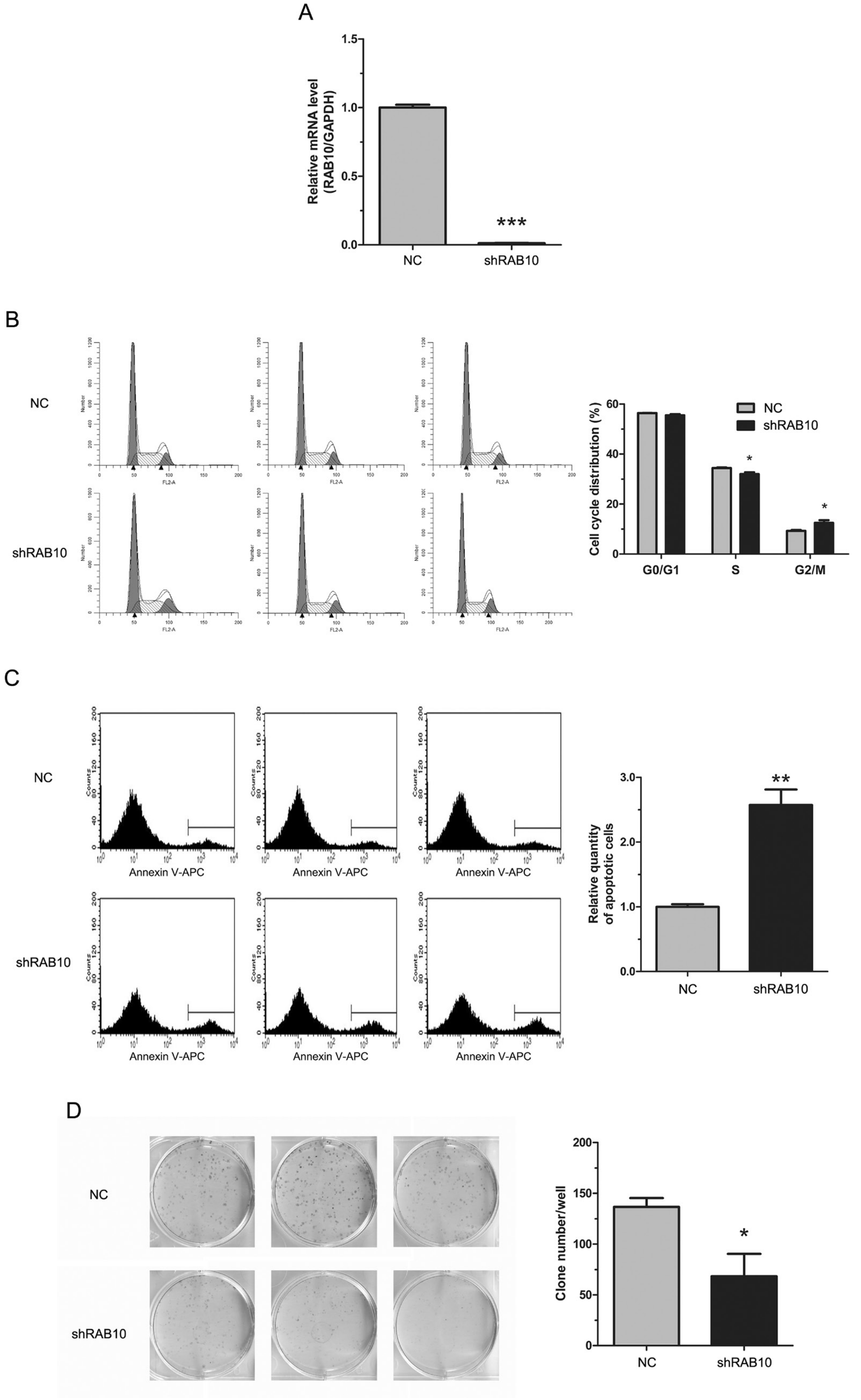


**Supplementary Figure 1: RAB10 knockdown induced cell cycle arrest and apoptosis and reduced colony formation in HepG2 cells.** Control-shRNA, cells infected with negative control shRNA lentivirus; shRAB10, cells infected with RAB10 shRNA lentivirus. (**A**) mRNA levels of RAB10 were knocked down efficiently by shRNA. (**B**) Knockdown of RAB10 expression induced G2/M phase arrest. (**C**) Knockdown of RAB10 increased apoptosis. (**D**) Knockdown of RAB10 significantly reduced colony formation. Data were shown as means ± SD ($n$ = 3), *$P$ < 0. 05, **$P$ < 0.01, ***$P$ < 0.001.

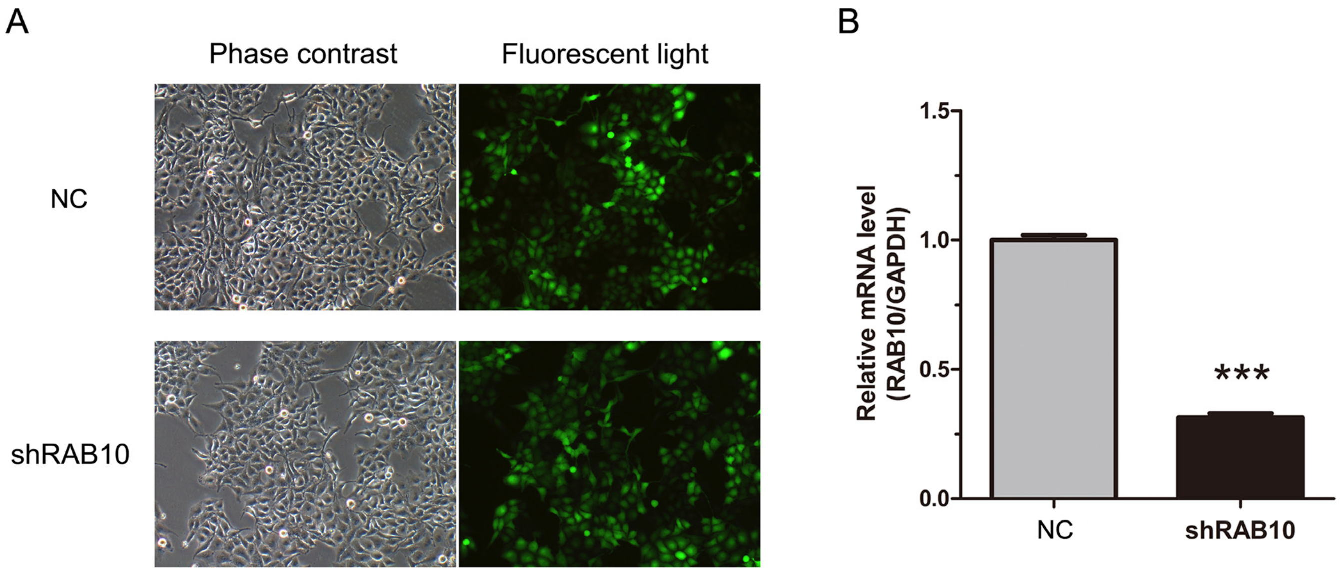


**Supplementary Figure 2: Efficiency of lentivirus infection.** shControl, human hepatocarcinoma cell line SMMC-7721 infected with control shRNA lentivirus; shRAB10, SMMC-7721 cells infected with RAB10 shRNA lentivirus. (**A**) SMMC-7721 cells were selected by puromycin for 48 h after infection, and examined by fluorescence and light microscopy. More than 85% of the cells expressed GFP (magnified 100×). (**B**) RAB10 mRNA levels were measured by qRT-PCR after infection and selection. Data were shown as means ± SD ($n = 3$), $***P < 0.001$.

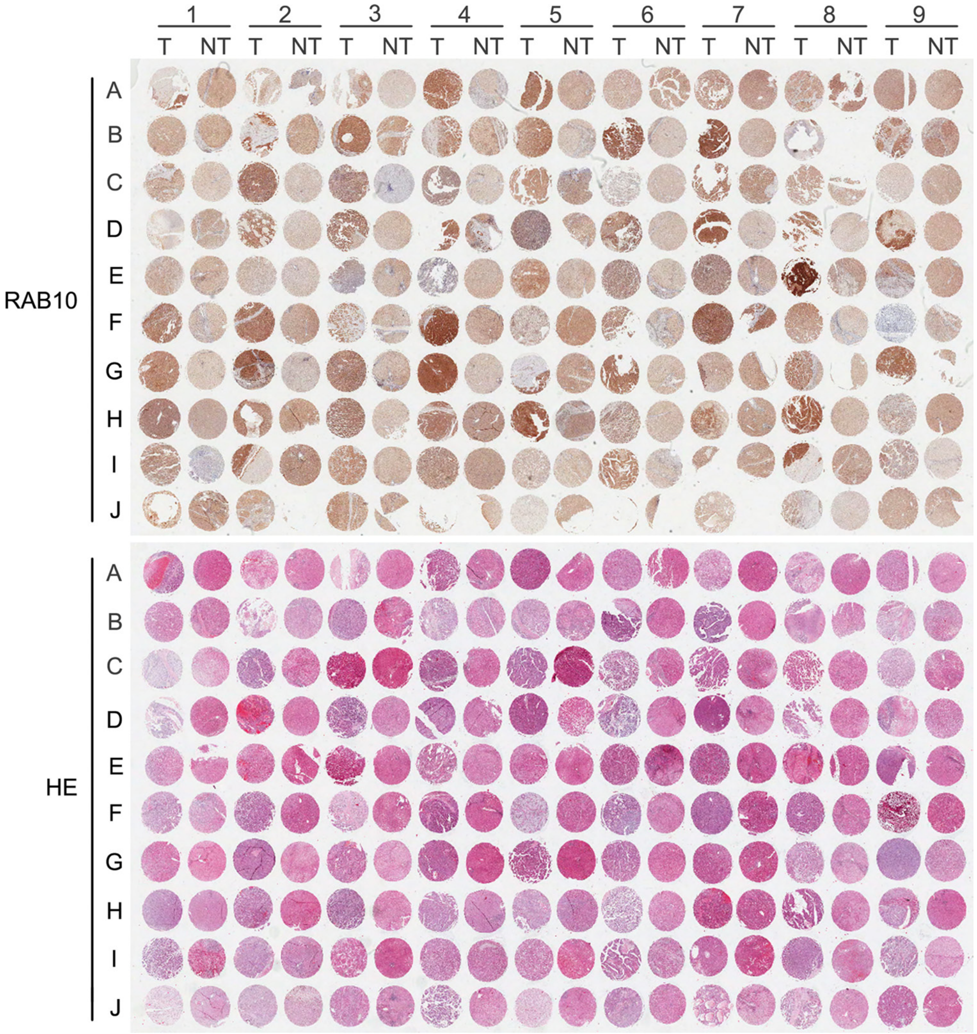


**Supplementary Figure 3: Expression of RAB10 was elevated in tissues of HCC patients.** Overview of immunohistochemical array for RAB10 expression in 90 pairs of tissues.
